# Intraspecific divergences and phylogeography of *Panzerina lanata* (Lamiaceae) in northwest China

**DOI:** 10.7717/peerj.6264

**Published:** 2019-01-24

**Authors:** Yanfen Zhao, Hongxiang Zhang, Borong Pan, Mingli Zhang

**Affiliations:** 1 Key Laboratory of Biogeography and Bioresource in Arid Land, Xinjiang Institute of Ecology and Geography, Chinese Academy of Sciences, Urumqi, Xinjiang, China; 2 University of Chinese Academy of Sciences, Beijing, China

**Keywords:** Intraspecific divergences, Aridification, Panzerina lanata, Desert expansion, Northwest China

## Abstract

Climatic fluctuations during the Quaternary significantly affect many species in their intraspecific divergence and population structure across northwest China. In order to investigate the impact of climate change on herbaceous plants, we studied *Panzerina lanata* (Lamiaceae), a widely distributed species. Sequences of two chloroplast DNA (cpDNA) intergenic spacers (*trnH-psbA* and *rpoB-trnC*) and a nuclear ribosomal region (nrDNA, ITS) were generated from 27 populations of *Panzerina lanata* and resulted in the identification of seven chloroplast haplotypes and thirty-two nuclear haplotypes. We applied AMOVA, neutrality test and mismatch distribution analysis to estimate genetic differentiation and demographic characteristics. The divergence times of the seven cpDNA haplotypes were estimated using BEAST. Our results revealed high levels of genetic diversity (cpDNA: *H*cp = 0.6691, *H*_T_ = 0.673; nrDNA: * H*nr = 0.5668*, H*_T_ = 0.577). High level of genetic differentiation (*G*_ST_ = 0.950) among populations was observed in the cpDNA sequences, while the genetic differentiation values (*G*_ST_ = 0.348) were low in nuclear sequences. AMOVA results revealed major genetic variation among the three groups: northern, central, and eastern group. However, the genetic differentiation in ITS data was not found. The species distribution modeling and demographic analysis indicated that *P. lanata* had not experienced recent range expansion. The occurrence of divergence between seven cpDNA haplotypes, probably during Pleistocene, coincides with aridification and expansion of the desert across northwest China that resulted in species diversification and habitat fragmentation. In addition, we discovered that the deserts and the Helan Mountains acted as effective geographic barriers that promoting the intraspecific diversity of *P. lanata*.

## Introduction

In China, studies on plant phylogeography have been mainly focusing on four regions: Qinghai-Tibet Plateau and Southwest China, West China, North and Northeast China, South and Southeast China (Liu et al., 2012; Qiu, Fu & Comes, 2011). The Qinghai-Tibet Plateau and southwest China are among the hotspots of biodiversity research in the world, which were not directly affected by ice shield. Most studies have shown that phylogeographic patterns were mainly influenced by climate fluctuations during the Quaternary, resulting in intraspecific divergences and regional range expansion (Guo et al., 2010; Liu et al., 2012). However, recent research on plant phylogeography in northwest China has expanded (Meng et al., 2015; Wang et al., 2016; Zhang et al., 2017), as its arid area includes not only Xinjiang but also Hexi Corridor, Qaidam Basin and western Helan Mountains (Dang & Pan, 2001). In these areas, the genetic structure and phylogeography of plants were mainly influenced by Quaternary climatic fluctuations (Meng et al., 2015; Wang et al., 2013). Climate change during the Pleistocene led to the low temperatures and aridification in northwest China, which promoted the desert expansion. Aridification and desert expansion have significant impacts on the phylogeography of many species in northwest China (Meng et al., 2015; Wang et al., 2016), such as allopatric divergences, speciation and habitat fragmentation of desert plants (Ma, Zhang & Sanderson, 2012; Meng & Zhang, 2011; Wang et al., 2016; Xu & Zhang, 2015a). In addition, geographical barriers (deserts, mountains, etc.) have resulted in segregation of species, limited seeds dispersal, and therefore rare genetic communication between populations, which lead to isolation and differentiation of genetic lineages of species (Cun & Wang, 2010; Liu et al., 2012; Wang et al., 2016). Research in these regions mainly concentrated on shrubs rather than herbaceous plants (Shi & Zhang, 2015; Su, Lu & Zhang, 2016; Su et al., 2015; Xu & Zhang, 2015a) that are more sensitive to climate oscillation. Here, we selected *Panzerina lanata* (Lamiaceae) as a suitable model to study the genetic structure of desert species in arid northwest China and its response to Quaternary climatic fluctuations.

The genus *Panzerina* (Lamioideae, Lamieae) contains two species (*Panzerina canescens and Panzerina lanata* ) that are mainly distributed in the desert and desert grassland areas of central Asia, and have been described in the Flora of China (Li & Hedge, 1994). *Panzerina lanata* is a perennial medicinal herb and widely distributed in the sandy desert steppes of Inner Mongolia, Gansu, Ningxia, and Shanxi. By contrast, *Panzerina canescens*, with a small population, is only found in the dry stony area of Xinjiang. Previous studies on *P. lanata* have mainly focused on biological characteristics (Cheryomushkina & Astashenkov, 2014; Li & Zhao, 2000), chromosome research (Li, Cao & Zhao, 1999), plant taxonomy and floristic analyses (Zhao et al., 1998; Zhao & Liu, 1997), other than its genetic diversity and phylogeography pattern.

Here, the phylogeographic structure of *P. lanata* was inferred by investigating two chloroplast DNA (cpDNA) intergenic spacers (*trnH-psbA* and *rpoB-trnC*) and a nuclear ribosomal region (nrDNA, ITS). Our objectives are to determine (1) the genetic diversity and structure of *P. lanata* and (2) how climatic fluctuations and geographical obstruction contribute to lineage differentiation of *P. lanata*.

## Materials and Methods

### Population sampling

Across northwest China, there are approximately 27 natural populations of *P. lanata*, from which we collected 269 individuals (Table 1). All individuals and 200 individuals from 27 natural populations were used for chloroplast analysis and nuclear gene analysis, respectively. In each population, 4–14 individuals were collected. To avoid duplicated sampling, we set 30 m as the minimum distance between individuals within each population. Fresh leaves were harvested and dehydrated using silica gel for later DNA isolation. Two specimens were collected from each population, and the voucher specimens were deposited in the Herbarium of Xinjiang Institute of Ecology and Geography, Chinese Academy of Science (XJBI). *Leonurus turkestanicus* and *Lagochilus ilicifolius* were included as outgroups in this analysis (Meng & Zhang, 2013).

**Table 1:** Detailed sampling sites, sample numbers, haplotype composition and genetic information from 27 populations of *Panzerina lanata* based on two cpDNA regions and one nrDNA analyses.

Region	Population code/location	Latitude/ Longitude (N/E)	Altitude (m)	N	Plastid haplotypes	H_cp_ (±SD)	*π*cp (±SD)	N	Nuclear haplotypes	H_nr_ (±SD)	*π*nr (±SD)
Total				269	7	0.6691 ± 0.0259	0.0077 ± 0.0039	200	32	0.5668 ± 0.0277	0.0018 ± 0.0013
North				26		0	0	–	–	–	–
	1 JLT	39.66°/105.70°	1032	5	H5	0	0	4	C2 C12 C13	0.6071 ± 0.1640	0.0021 ± 0.0016
	2 SZ	41.38°/107.04°	1,586	11	H5	0	0	11	C1 C2 C6 C17 C18 C19 C20 C21	0.8918 ± 0.0328	0.0059 ± 0.0035
	3 BYT1	41.52°/106.91°	1,622	10	H5	0	0	10	C1 C2 C20 C22	0.6789 ± 0.0742	0.0023 ± 0.0016
Central				89		0.6673 ± 0.0177	0.0008 ± 0.0005	–	–	–	–
	4 ALZQ	38.16°/107.59°	1,344	10	H3	0	0	7	C2	0	0
	5 BYHT	38.93°/105.78°	1,660	12	H2 H4	0.1667 ± 0.1343	0.0016 ± 0.0011	7	C2 C6 C7 C8 C9 C10 C11	0.8791 ± 0.0576	0.0031 ± 0.0021
	6 HST	38.70°/105.57°	1,366	11	H2	0	0	10	C2 C24	0.1000 ± 0.0880	0.0003 ± 0.0004
	7 ND	38.08°/106.72°	1,342	10	H3	0	0	6	C2 C5 C31	0.4394 ± 0.1581	0.0007 ± 0.0007
	8 JT	37.38°/104.60°	1,586	10	H7	0	0	9	C2	0	0
	9 GL	37.64°/103.18°	1,741	13	H7	0	0	10	C2	0	0
	10 SH	37.85°/105.33°	1,408	14	H7	0	0	13	C2 C31	0.0769 ± 0.0697	0.0001 ± 0.0002
	11 BTG	37.86°/106.30°	1,364	9	H3	0	0	7	C2 C5	0.3626 ± 0.1302	0.0005 ± 0.0006
East				154		0.1364 ± 0.0373	0.0017 ± 0.0010	–	–	–	–
	12 HQH	39.35°/109.57°	1,433	10	H1 H2	0.2000 ± 0.1541	0.0021 ± 0.0013	5	C1 C2	0.2000 ± 0.1541	0.0003 ± 0.0004
	13 YJHL1	39.36°/109.79°	1,352	8	H1	0	0	8	C1 C2	0.4000 ± 0.1135	0.0006 ± 0.0006
	14 YJHL2	39.26°/109.80°	1,336	4	H1	0	0	4	C1 C3	0.4286 ± 0.1687	0.0006 ± 0.0007
	15 TK	39.19°/109.41°	1,377	10	H1	0	0	8	C2 C4	0.2333 ± 0.1256	0.0003 ± 0.0005
	16 KN	39.47°/108.37°	1,356	8	H1	0	0	5	C2 C5	0.3556 ± 0.1591	0.0005 ± 0.0006
	17 XN	39.77°/108.65°	1,423	8	H1	0	0	5	C1 C2 C5	0.7111 ± 0.0860	0.0014 ± 0.0012
	18 DGTL	40.49°/109.02°	1,267	10	H1	0	0	5	C2	0	0
	19 EGB	40.43°/109.28°	1,020	9	H1	0	0	8	C2 C14 C15 C16	0.6000 ± 0.1254	0.0025 ± 0.0017
	20 BYT2	41.71°/107.00°	1,394	10	H5 H6	0.5333 ± 0.0947	0.0130 ± 0.0071	10	C1 C2 C20 C23	0.6895 ± 0.0782	0.0022 ± 0.0015
	21 XJW	39.96°/111.02°	1,316	13	H1	0	0	8	C1 C2 C25	0.7000 ± 0.0506	0.0014 ± 0.0012
	22 ELT	38.97°/109.90°	1,251	11	H1	0	0	8	C1 C2 C26	0.6333 ± 0.0737	0.0016 ± 0.0013
	23 BJ	38.06°/109.67°	1,047	11	H1	0	0	6	C2	0	0
	24 MH	38.49°/109.48°	1,222	11	H1	0	0	6	C2 C27	0.3030 ± 0.1475	0.0005 ± 0.0006
	25 YQP	37.64°/108.91°	1,312	10	H1	0	0	5	C2	0	0
	26 DB	37.59°/107.54°	1,371	9	H1	0	0	8	C1 C2 C5 C28 C29 C30	0.7167 ± 0.0988	0.0026 ± 0.0018
	27 WST	39.42°/106.63°	1,320	12	H1	0	0	7	C2 C32	0.1429 ± 0.1188	0.0002 ± 0.0003

### DNA isolation, PCR amplification, and sequencing

CTAB method was used for genomic DNA isolation (Doyle & Doyle, 1987). The cpDNA intergenic spacers*, trnH-psbA* and *rpoB-trnC* (Sang, Crawford & Stuessy, 1997; Shaw, Lickey & Beck, 2005), and a nuclear ribosomal region (ITS1-ITS4) were amplified. The PCR reactions (25 µL) contained 10 ×PCR buffer (2.5 µL), MgCl_2_ (25 mM, 2.5 µL), dNTP mixture (2.5 mM, 2.0 µL), forward primer (1 µL) and reverse primer (1 µL), *Taq* polymerase (0.125 µL) (Takara, Kusatsu, Japan), and genomic DNA (1 µL) as template. The amplification cycles were as following: initial denaturation (95 °C, 4 min), 36 cycles of denaturation (94 °C, 30s), annealing (52 °C, 30s) and extension (72 °C, 1 min), and a final extension (72 °C, 10 min). The PCR products were examined by electrophoresis using agarose gel (1%), isolated with a QIAquick Gel Extraction Kit (Qiagen, Hilden, Germany), and sequenced on ABI Prism 3730 Genetic Analyzer (Sangon, Shanghai, China).

For DNA sequence editing, we used SeqMan (Lasergene, USA), and used BioEdit (Hall, 1999) for alignment. All sequences are deposited in GenBank under accession numbers MK299519–MK299550 for ITS, MK299551–MK299556 for trnH-psbA, MK299557–MK299559 for rpoB-trnC.

### Genetic diversity and population structure

Arlequin 3.5 was applied to determine haplotype diversity (*H*) and nucleotide diversity (*π*) (Excoffier & Lischer, 2010). For subdivision of their geographical structure, we used SAMOVA v1.0 (Dupanloup, Schneider & Excoffier, 2002). We set 2 ≤ *K* ≤ 12 until the *F*_CT_ values reached the maximum and, when a single population was clustered into one group, the combination was excluded (Beatty, Provan & Comes, 2013; Iwasaki et al., 2012). With Arlequin 3.5, AMOVA estimated genetic variation on three different levels: inter-groups, inter-populations within groups, and intra-populations. Calculation of genetic differentiation was determined using a significance test based on 1,000 permutations. To identify genetic differentiation (*G*_ST_, *N*_ST_), average heterozygosity within populations (*H*_S_) and across total populations (*H*_T_), we used Permut v1.0 (Pons & Petit, 1996) for 1,000 permutation tests. *H*_T_ and *H*_S_ were used to estimate genetic diversity. These two parameters (*N*_ST_, *G*_ST_) were used to test if phylogeographic structure existed. By applying the median-joining algorithm, we also implemented the phylogenetic relationship among the haplotypes Network v5.0 (Bandelt, Forster & Röhl, 1999).

### Demographic history and divergence time analyses

In order to test whether all populations and groups of *P. lanata* divided by the SAMOVA experienced demographic expansion, we used Arlequin 3.5 to determine Tajima’s *D* (Tajima, 1989) and Fu’s *F*_*S*_ (Fu, 1997) with 1,000 permutation tests. We further applied a mismatch distribution analysis to measure population expansion for all groups. In general, unimodal distribution patterns showed a recent expansion event, while the distribution pattern was stable according to bimodal and multimodal results. We also estimated the sum of squared deviations (SSD) and the raggedness index of Harpending (HRag) (Harpending, 1994) between the observed and expected mismatches in Arlequin 3.5 with 1,000 permutation tests. The significance of *P* was examined if the populations experienced expansion.

We estimated the divergence time between different lineages of *P. lanata* using Beast v1.6.1 (Drummond & Rambaut, 2007). As most angiosperms have a cpDNA nucleotide substitution rate of 1.0–3. 0 × 10^−9^ s/s/y, we use 2. 0 × 10^−9^ s/s/y with a SD of 6. 080 × 10^−10^ s/s/y to estimate the divergence time of these two cpDNA regions (*trnH-psbA* and *rpoB-trnC*) (Jia et al., 2012; Wang et al., 2016). We ran the GTR+G substitution model and the MCMC chains for 10,000,000 generations and sampled every 1,000 generations. The effective population sizes (ESS > 200) were checked in Trace v1.5. We used TreeAnnotator v1.6.1 to implement the maximum clade credibility tree with a burn-in of 1,000 trees. For tree editing, we applied FigTree v1.3.1.

### Species distribution inference

For estimation of the past (Last Glacial Maximum, 21,000 before present) and present distribution ranges of *P. lanata*, we used the ecological niche model to infer the potential distribution area. We simulated the potential distribution with the maximum entropy method in MAXENT 3.3.1 (Phillips, Anderson & Schapire, 2006). Geographical information of *P. lanata* was gleaned from field collection records and the Chinese Virtual Herbarium (http://www.cvh.org.cn/). We used 36 points in these modeling analyses (Fig. S1). For estimation of current potential distribution, 19 bioclimatic variables in the WorldClim database (http://www.worldclim.org/download) were applied. To simulate the distribution during Last Glacial Maximum, we followed the Model for Interdisciplinary Research on Climate (Hasumi & Emori, 2004) and the Community Climate System Model (Collins et al., 2006). For the model and the training data, we used the AUC value to estimate the goodness of fit.

## Results

### Sequence differences and haplotype patterns of cpDNA

After alignment, the *rpoB-trnC* and *trnH-psbA* sequences were 1,145 bp and 367 bp respectively, and 1,512 bp in total. In the combined data, we identified seven haplotypes (H1–H7) (Table 1) and 17 polymorphic sites (14 substitutions and three indels) from 269 individuals collected from 27 populations (Table 2). The total haplotype diversity (*H*_cp_) was 0.6691 with a within-population variation of 0–0.5333 while the overall nucleotide diversity (*πcp*) was 0.0077 with a range of 0–0.0130. The BYT2 population had higher *H* and *π* values than others (Table 1).

**Table 2:** Variation sites of each haplotype in two cpDNA sequences of *Panzerina lanata*.

Sequence position																			
																	1		
																	3		
						4		7									9		
						0		5									4-		
						7-		4-				1	1	1	1	1	1	1	1
					1	4	4	7	7	8	8	2	2	2	2	3	4	4	4
			5	7	7	2	7	5	8	4	5	1	2	2	4	3	1	3	5
			2	1	6	0	4	9	7	3	4	5	0	7	9	2	4	9	6
Haplotype																			
H1			C	T	T	△	A	–	A	C	G	G	T	A	A	A	–	A	G
H2			A	T	T	–	A	–	A	C	G	G	T	C	A	A	–	A	G
H3			A	T	T	–	A	–	A	C	G	G	C	C	A	A	–	A	A
H4			C	T	T	△	A	–	A	C	G	G	T	C	A	A	–	A	G
H5			C	G	G	△	C	□	C	T	T	A	T	A	T	T	–	G	G
H6			C	T	T	△	A	–	A	C	G	G	T	A	A	A	∘	A	G
H7			A	T	T	–	A	–	A	C	G	G	C	C	A	A	–	A	G

**Notes.**

△ TATTTAGATATGTT □ TTTTCT ∘ TATTTATCTATTCTATTTTCA

**Figure 1:**
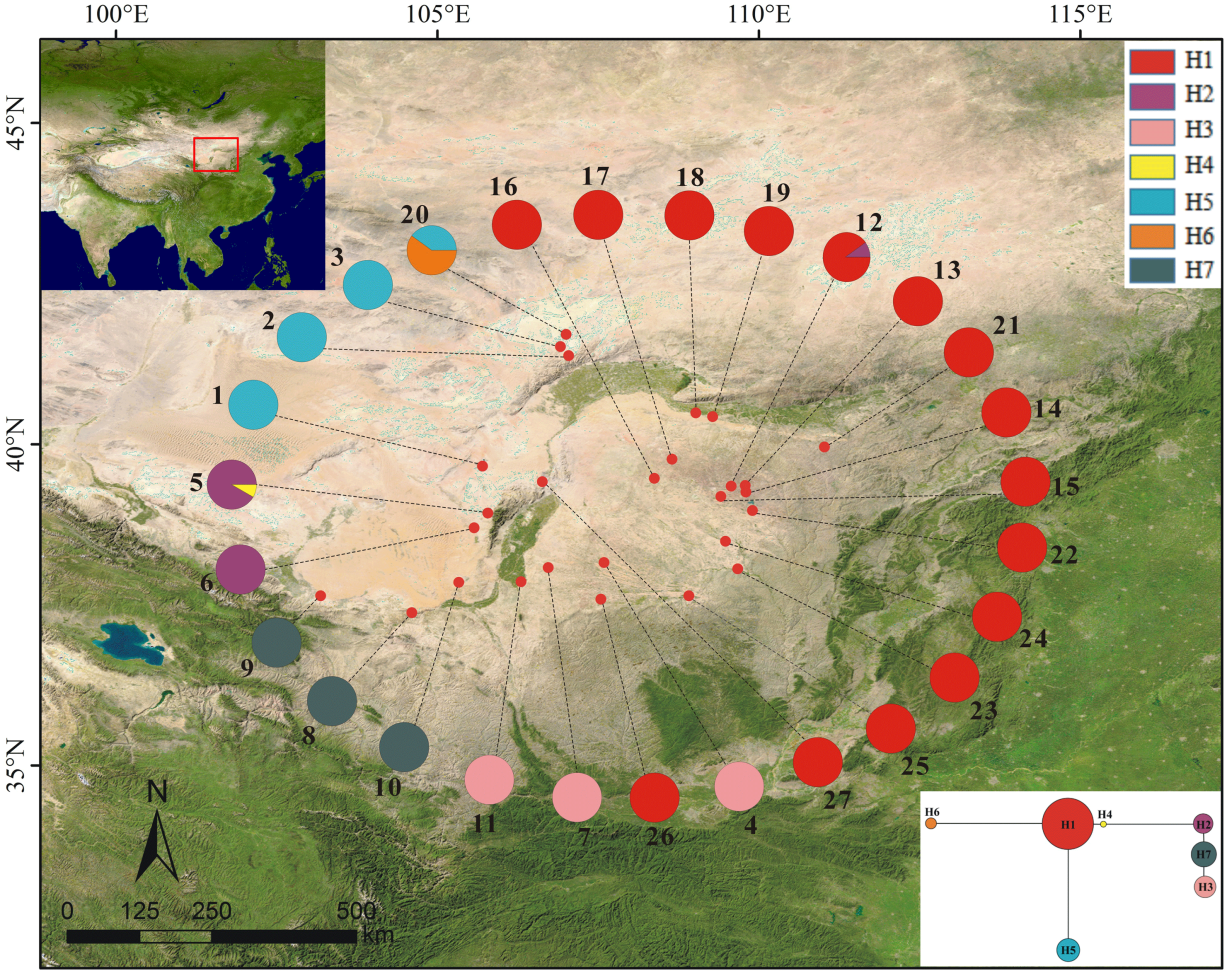
Sample locations (population names as shown in Table 1), geographic distribution and relationships of cpDNA haplotypes (H1–H7) from 27 populations of *Panzerina lanata* in northwestern China.

Among the 27 populations examined, only three of them had two haplotypes and all others a single haplotype. In the seven haplotypes identified among *P. lanata* samples, H1 was widely distributed and was the only haplotype in most populations. H5 was found in four populations, three of which were “H5-only” ones. H2, H3, and H7 were exclusively detected in three different populations. H4 and H6 were specific haplotypes found in BYHT and BYT2, respectively (Table 1; Fig. 1).

### Sequence differences and haplotype patterns of ITS

The attempts for amplification and sequencing of ribosomal DNA from several individuals in each of the 27 populations failed. Two hundred individuals were successfully sequenced. The total length was 635 bp, and thirty-two haplotypes (C1-C32) were obtained (Table 1). The total haplotype diversity (*H*_nr_) was 0.5668, with a range of 0.0769–0.8918, whereas the nucleotide diversity (*πnr*) was 0.0018, with a range of 0.0001–0.0059. Populations JLT, SZ, BYT1, BYHT, XN, EGB, BYT2, XJW, ELT and DB had high *H* and *π* values. Most populations had Haplotype C1 or C2.

### Genetic diversity and population structure

SAMOVA analysis divided all populations of *P. lanata* into three groups based on the cpDNA analysis: (1) the northern group (populations 1–3), (2) the central group (populations 4–11), and (3) the eastern group (populations 12–27).

Among all of the populations, the genetic diversity was high (cpDNA: *H* cp = 0.6691 ± 0.0259, *H*_T_ = 0.673 ±0.0869; nrDNA: *H* nr = 0.5668 ± 0.0277, *H*_T_ = 0.577 ± 0.0685), while the average within-population genetic diversity was relatively low in the cpDNA (*H*_S_ = 0.033 ±  0.0214) (Table 3). The cpDNA showed high levels of inter-population genetic diversity (*G*_ST_ = 0.950 ± 0.0308, *N*_ST_ = 0.922 ± 0.0559), suggesting that genetic differentiation mainly occurred among populations. However, as indicated by the nrDNA sequences, the genetic differentiation among the populations (*G*_ST_ = 0.348 ±  0.0562) was low. In either cpDNA or nrDNA, *N*_ST_ and *G*_ST_ showed no significance regarding their difference (*p* > 0.05), thus no clear phylogeographic structure is present in *P. lanata*. In the central group, the genetic diversity was high (*H* cp = 0.6673 ±  0.0177, *H*_T_ = 0.753 ± 0.0476), with a low average intra-population genetic diversity (*H*_S_ = 0.021 ± 0.0208). The level of genetic differentiation among populations was high (*G*_ST_ = 0.972 ± 0.0263, *N*_ST_ = 0.755 ±  0.1630). By contrast, both the genetic diversity (*H* cp = 0.1364 ± 0.0373, *H*_T_ = 0.137 ±  0.1122) and the average within-population genetic diversity (*H*_S_ = 0.046 ± 0.0348) of the eastern group were low (Table 3). Of the total variation, 89.87% (*p* < 0.001) occurred in the cpDNA sequences among northern, central, and eastern groups, 5.22% was inter-populations variation within groups, and 4.91% was intra-populations variation (Table 4). This result of the hierarchical analysis was consistent with the *F*_ST_ (0.9509, *p* < 0.001) and *F*_CT_ (0.8987, *p* < 0.001) values. However, genetic variation in the nrDNA mainly occurs within populations (*F*_Sc_ = 0.30026; *F*_CT_ = 0.08700) (Table 4).

**Table 3:** Estimates of genetic diversity (H_*S*_, H_*T*_) and genetic differentiation (G_*ST*_ , N_*ST*_) of *Panzerina lanata* based on two cpDNA and one nrDNA sequences data.

Markers	Region	*H*_*S*_	*H*_*T*_	*G*_*ST*_	*N*_*ST*_
cpDNA	Total	0.033(0.0214)	0.673(0.0869)	0.950(0.0308)	0.922(0.0559)
	Central	0.021(0.0208)	0.753(0.0476)	0.972(0.0263)	0.755(0.1630)
	East	0.046(0.0348)	0.137(0.1122)	0.665(NC)	0.443(NC)
ITS	Total	0.376(0.0578)	0.577(0.0685)	0.348(0.0562)	0.355(0.0524)

**Table 4:** Results of analysis of molecular variance (AMOVA) of *Panzerina lanata* using two cpDNA regions and one nrDNA sequence.

Markers	Grouping of regions	Source of variation	*d.f.*	*SS*	*VC*	*PV(%)*	Fixation index
		Among groups	2	1,327.772	8.84162	89.87	*F*_CT_ = 0.8987**
	Total	Among populations within groups	24	133.813	0.51320	5.22	*F*_SC_ = 0.5150**
		Within populations	242	116.950	0.48326	4.91	*F*_ST_ = 0.9509**
cpDNA		Total	268	1,578.535	9.83808		
		Among populations	7	37.688	0.47011	73.47	
	Central	Within populations	81	13.750	0.16975	26.53	*F*_ST_ = 0.7347**
		Total	88	51.438	0.63987		
		Among populations	15	96.125	0.58979	44.09	
	East	Within populations	138	103.200	0.74783	55.91	*F*_ST_ = 0.4409**
		Total	153	199.325	1.33762		
		Among groups	2	19.265	0.05360	8.7	*F*_SC_ = 0.30026**
ITS	Total	Among populations within groups	24	68.320	0.16888	27.41	*F*_ST_ = 0.36113**
		Within populations	373	146.802	0.39357	63.89	*F*_CT_ = 0.08700*
		Total	399	234.387	0.61605	

### Population divergence and demography: cpDNA analysis

**Figure 2:**
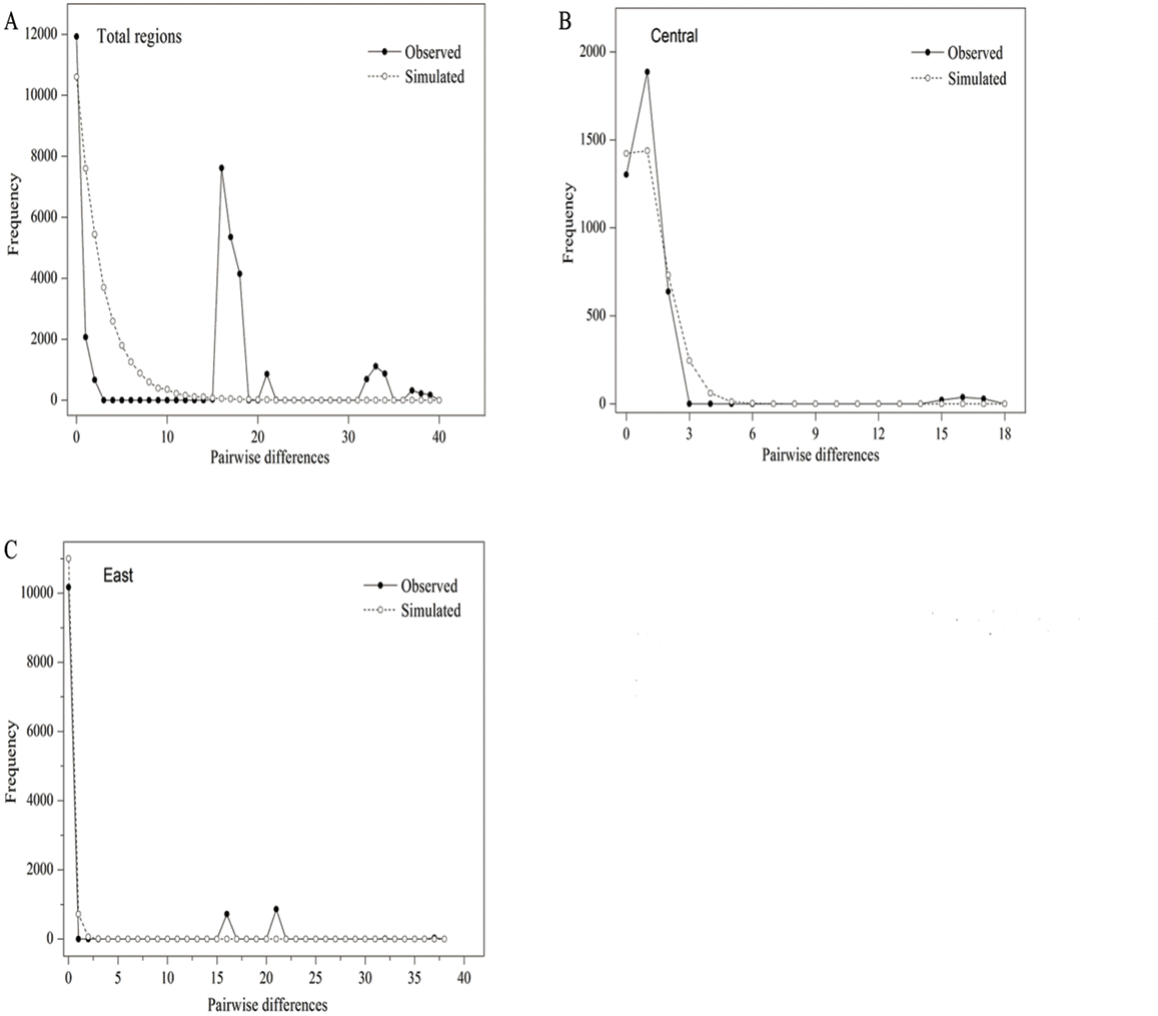
Mismatch distribution analysis of all populations (A), central group (B) and eastern group (C) from two cpDNA regions.

The parameters of Tajima’s *D* and Fu’s *F*_*S*_ (*p* > 0.05) were positive and insignificant, indicating that *P. lanata* has not experienced a recent expansion (Table 5; Fig. 2A). In the central group, the Tajima’s *D* and Fu’s *F*_*S*_ (*p* > 0.05) were insignificant and positive, indicating that the group has not experienced recent expansion. This conclusion was also confirmed by the *P*-values (*p* < 0.05) of SSD, Hrag, and the multimodal mismatch analysis (Table 5; Fig. 2B). In the eastern group, the parameter of Tajima’s *D* (−1.8814, *p* < 0.05 ) was significantly negative, with the *P*-values of SSD and Hrag were both greater than 0.05. In addition, the mismatch distribution was unimodal, indicating that the populations had experienced a regional-scale expansion (Table 5; Fig. 2C). In the northern group, H5 was the only haplotype, and therefore it was impossible to analyze its population expansion further.

**Figure 3:**
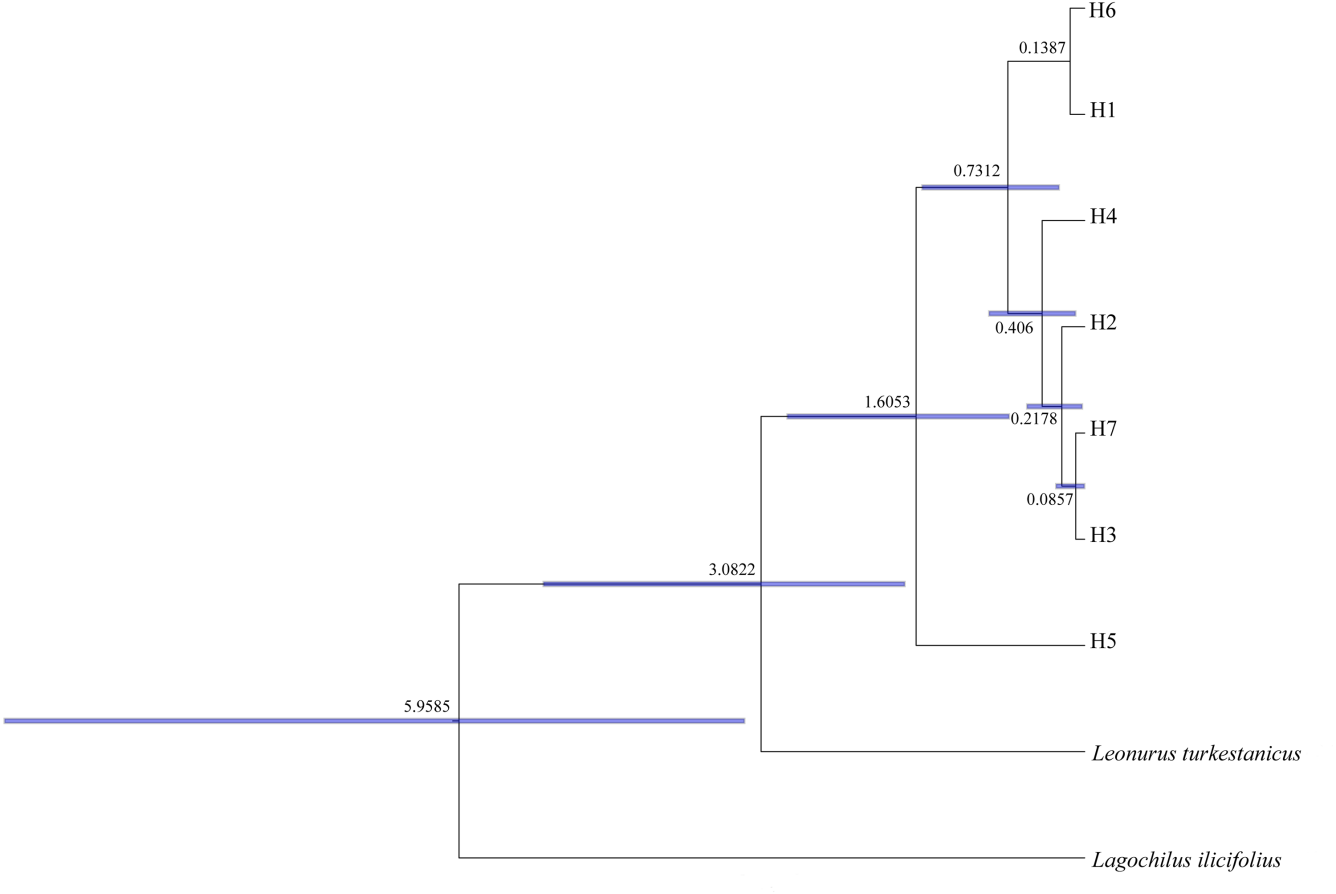
Divergence time estimated of 7 cpDNA haplotypes of *Panzerina lanata* based on BEAST analysis.

Meanwhile, Beast analysis unraveled that the occurrence time of divergence between these seven haplotypes was from the early Pleistocene (1.6053; 95% HPD: 0.7215–2.8307) Mya to the late Pleistocene (0.0857; 95% HPD: 0.0033–0.2721) Mya (Fig. 3).

**Table 5:** Results of the neutrality test (Tajima’s D , Fu’s Fs) and mismatch distribution analysis for the whole populations, central and eastern group of *Panzerina lanata*.

Markers	Region	Tajima’s D(*P* value)	Fu’s Fs(*P* value)	SSD (*P* value)	Hrag(*P* value)	Mismatch distribution
cpDNA	Total	1.2613(0.9220)	30.3737(1)	0.1433(0.0800)	0.1417(0.2470)	Multimodal
	Central	0.8089(0.8020)	1.877(0.8430)	0.0188(0.0220)	0.1506(0)	Multimodal
	East	−1.8814(0.0030)	7.0559(0.9630)	0.0176(0.0540)	0.7637(0.7840)	Unimodal

### Species expansion trends: current and future distribution

**Figure 4:**
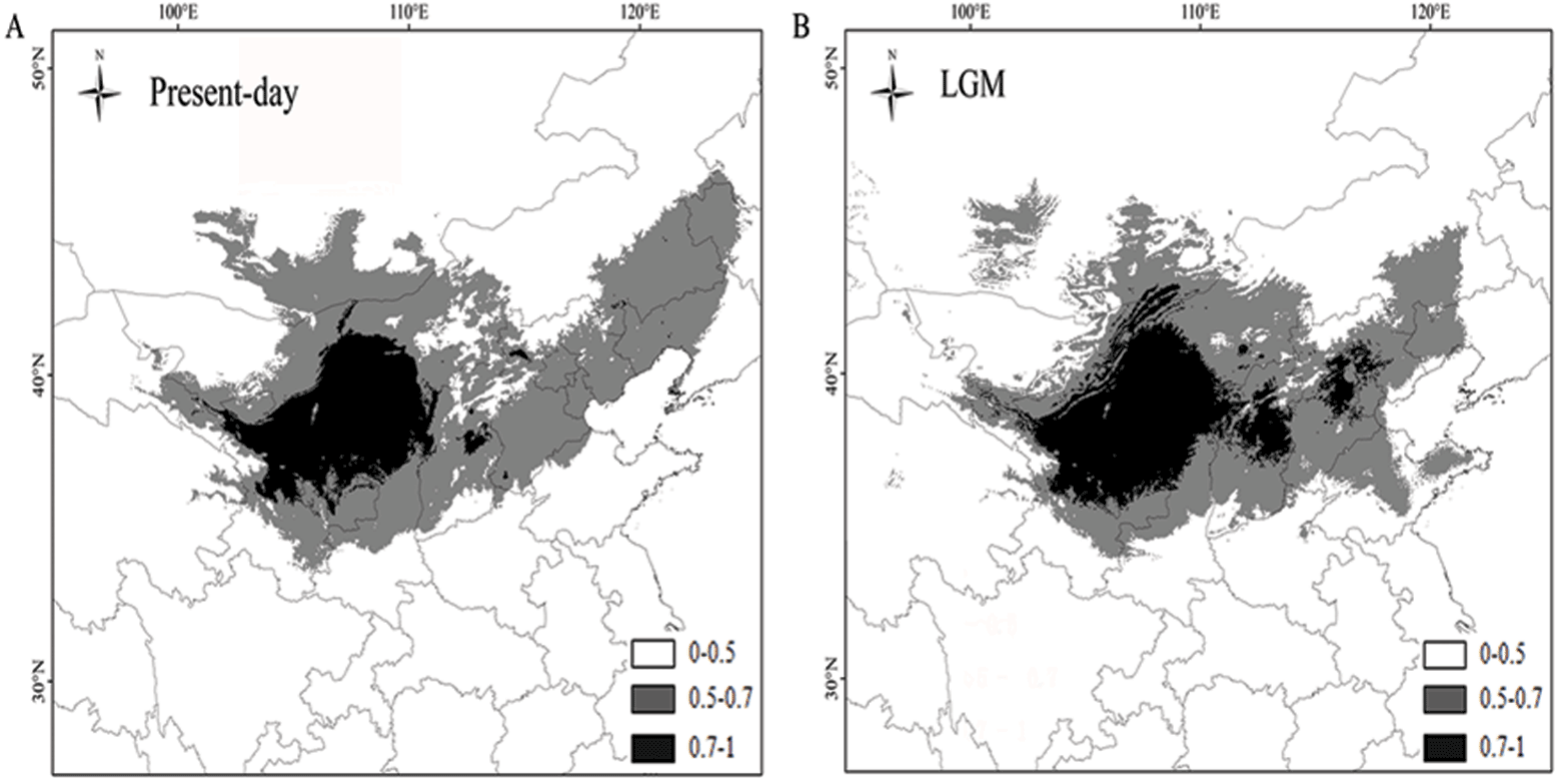
Maps depicting potential distribution of *Panzerina lanata* in northwest China during present-day ** (A) and LGM (B) based on the MIROC model.

The AUC values of *P. lanata* were 0.997/0.997 (the current model) and 0.997/0.997 (under the MIROC climate model). The higher AUC values indicated that the model was more suitable for the current distribution and the potential distribution during the LGM. The results revealed that the current distribution of *P. lanata* has not experienced recent expansion. The central region remained stable, whereas the eastern region showed an expanded distribution pattern (Figs. 4A, 4B).

## Discussion

### Genetic diversity of *P. lanata*

We found the high genetic diversity in *P. lanata.* The value of genetic differentiation (*G*_ST_ = 0.950) was higher than the average reported for other angiosperms (*G*_ST_ = 0.637) (Petit et al., 2005), indicating a stronger inter-population differentiation of the cpDNA sequences in *P. lanata*. The total genetic diversity of *P. lanata* (*H*_T_ = 0.673) was similar to that of *Allium mongolicum* (*H*_T_ = 0.693) but lower than that of *Lagochilus ilicifolius* (*H*_T_ = 0.925), both of which are herbaceous plants in northwest China. The average within-population genetic diversity (*H*_S_ = 0.033) of *P. lanata* was lower than *Allium mongolicum* (*H*_S_ = 0.180) (Meng & Zhang, 2011; Zhang et al., 2017), mainly due to its morphology and the influence of gravity on seed dispersal. Since the seeds can only be spread over short distances, a severely restricted gene flow between populations was resulted (Meng & Zhang, 2011). Concerning the nuclear gene sequence, the genetic diversity (*H*_T_ = 0.577, *H*_S_ = 0.376) was higher due to the long distance of pollen transmission. High genetic variation and unique haplotypes are usually associated with centers of plant diversity or potential refugia, whereas regions of recent colonization have low levels of genetic variation (Li et al., 2010; Meng & Zhang, 2011; Stewart et al., 2010). The central group had the highest genetic diversity. The Helan Mountains is a diversity center for species, and a high level of genetic diversity is expected. Such diversity centers get support from other species distributed in the area (Meng & Zhang, 2011; Shi & Zhang, 2015). In the eastern group, haplotype H1 was widely distributed in most populations and the most haplotypes for all populations except HQH and BYT2. During the range expansion process, the founder effect should be responsible for less genetic diversity, often causing a single prevailing haplotype (Hewitt, 2000; Xu & Zhang, 2015b; Zhang, Volis & Sun, 2010). Therefore, the low genetic diversity in the eastern group should be attributed to the founder effect.

### Intraspecific divergence across DNA sequences

All populations of *P. lanata* were divided into three groups. In the eastern group, the haplotype H1 was dominant, and H2, H5, and H6 were rare haplotypes. The central group was dominated by the haplotypes H2, H3, and H7, and H4 was rare. The northern group only had the haplotype H5. The haplotypes were unique among the three groups except for H2 and H5, suggesting a division between the three groups. AMOVA analyses showed that the variation among the three groups contributed to the total variation, indicating the presence of inter-group restricted gene flow. The occurrence of divergence among *P. lanata* populations was in the early to late Pleistocene, as the aridification and desert expansion also occurred during Pleistocene in northwest China. Therefore, we speculate that this species showed a diversification distribution, which may be attributed to the aridification in the early Pleistocene. In addition, the northern and eastern groups are separated by the desert (Hobq Desert, Mu Us Sandy Land, and Ulan Buh Desert), the geographical barriers that may limit the gene flow among the three groups, causing genetic differentiation within the species. The central group is located around the Helan Mountains. Previous studies have shown that the Helan Mountains acted as migration corridors for recolonization after ice ages as well as a geographical barrier (Meng et al., 2015; Zhang et al., 2017). Therefore, we speculate that deserts and the Helan Mountains may act as geographical barriers restricting the long-distance dispersal of *P. lanata* seeds, eventually resulting in isolation and differentiation of the populations in these three groups (northern, eastern, and central groups). Nuclear gene data showed that low genetic differentiation among the three groups. Because pollen transmission was far away, geographical barriers (deserts) did not hinder the gene flow among the three groups.

### Demographic history of *P. lanata*

According to the neutrality test and mismatch analysis, no expansion event has occurred in this species recently, except the eastern group. Previous studies have shown that plants have lower genetic diversity and a single, widely distributed haplotype in regions with rapid expansion (Hewitt, 1996; Shi & Zhang, 2015; Zhang & Zhang, 2012). The eastern group has a low number of haplotypes and limited genetic diversity. H1 is widely distributed and is the only haplotype for most populations in the eastern group, indicating that the region has experienced an expansion. The mismatch analysis and neutrality test also confirmed these results. In comparison to the LGM distribution of *P. lanata*, the central region showed a stable distribution pattern, whereas the eastern region showed an expanding distribution. This may be due to the high annual precipitation in the central region and eastern region allowing for more suitable habitat for *P. lanata*.

## Conclusions

Our results show that the *P. lanata* recently has not experienced range expansion. Both chloroplast data and nuclear gene data showed high genetic diversity. Because of the different pollination mechanisms, the gene flow of cpDNA is mainly mediated by seeds while the nuclear DNA by both seeds and pollen. The chloroplast data indicated that the variation occurred mainly among three groups. Aridification and geographical isolation (Deserts and Helan Mountains) limited gene flow among populations and played critical roles in affecting genetic diversity and genetic differentiation of *P. lanata*. According to our nuclear gene data, the geographical isolation such as desert and Mountains had less influence on the differentiation of *P. lanata* due to the ability of pollens for longer distance traveling.

##  Supplemental Information

10.7717/peerj.6264/supp-1 Figure S1 The geographical information (include the latitude and longitude of the simulated distribution area ) of *P. lanata* was gleaned from field collection records and the Chinese Virtual Herbarium

## Additional Information and Declarations

### Data Deposition

The following information was supplied regarding data availability: The raw data is available as a Supplemental File.
